# The Glucose Transporter 5 Enhances CAR-T Cell Metabolic Function and Anti-tumour Durability

**DOI:** 10.21203/rs.3.rs-4342820/v1

**Published:** 2024-05-07

**Authors:** Bakir Valentić, Andre Kelly, Alexander A. Shestov, Zhiyang Gan, Feng Shen, Adam Chatoff, Alison Jaccard, Claudia V. Crispim, John Scholler, Simon Heeke, Nathaniel W. Snyder, Saba Ghassemi, Nicholas Jones, Saar Gill, Roddy S. O’Connor

**Affiliations:** 1 Center for Cellular Immunotherapies, Perelman School of Medicine at the University of Pennsylvania, Philadelphia, PA 19104, USA; 2 Department of Pathology and Laboratory Medicine, Perelman School of Medicine at the University of Pennsylvania, Philadelphia, PA 19104, USA; 3 Division of Haematology-Oncology, Perelman School of Medicine at the University of Pennsylvania, Philadelphia, PA 19104, USA; 4 Aging + Cardiovascular Discovery Center, Department of Cardiovascular Sciences, Lewis Katz School of Medicine at Temple University, Philadelphia, PA 19140, USA; 5 Department of Thoracic/Head & Neck Medical Oncology, The University of Texas MD Anderson Cancer Center, Houston, TX, USA; 6 Institute of Life Science, Swansea University Medical School, Swansea SA2 8PP, UK

## Abstract

Activated T cells undergo a metabolic shift to aerobic glycolysis to support the energetic demands of proliferation, differentiation, and cytolytic function. Transmembrane glucose flux is facilitated by glucose transporters (GLUT) that play a vital role in T cell metabolic reprogramming and anti-tumour function. GLUT isoforms are regulated at the level of expression and subcellular distribution. GLUTs also display preferential selectivity for carbohydrate macronutrients including glucose, galactose, and fructose. GLUT5, which selectively transports fructose over glucose, has never been explored as a genetic engineering strategy to enhance CAR-T cells in fructose-rich tumour environments. Fructose levels are significantly elevated in the bone marrow and the plasma of acute myeloid leukaemia (AML) patients. Here, we demonstrate that the expression of wild-type GLUT5 restores T cell metabolic fitness in glucose-free, high fructose conditions. We find that fructose supports maximal glycolytic capacity and ATP replenishment rates in GLUT5-expressing T cells. Using steady state tracer technology, we show that ^13^C_6_ fructose supports glycolytic reprogramming and TCA anaplerosis in CAR-T cells undergoing log phase expansion. In cytotoxicity assays, GLUT5 rescues T cell cytolytic function in glucose-free medium. The fructose/GLUT5 metabolic axis also supports maximal migratory velocity, which provides mechanistic insight into why GLUT5-expressing CAR-Ts have superior effector function as they undergo “hit-and-run” serial killing. These findings translate to superior anti-tumour function in a xenograft model of AML. In fact, we found that GLUT5 enhances CAR-T cell anti-tumour function *in vivo* without any need for fructose intervention. Accordingly, we hypothesize that GLUT5 is sufficient to enhance CAR-T resilience by increasing the cells’ competitiveness for glucose at physiologic metabolite levels. Our findings have immediate translational relevance by providing the first evidence that GLUT5 confers a competitive edge in a fructose-enriched milieu, and is a novel approach to overcome glucose depletion in hostile tumour microenvironments (TMEs).

## Introduction

Chimeric antigen receptors (CARs) provide an important approach to redirect T cell specificity against cancer.^1^ CARs reprogram T cell metabolism indirectly via their built-in co-stimulatory domains, culminating in T cell progeny with enhanced persistence and anti-tumour durability.^2^ Direct metabolic reprogramming via armour CAR strategies and/or conditioning approaches has immediate translational relevance for adoptive immunotherapies. In the clinical sector, CAR-Ts are expanded for 9–14 days prior to adoptive transfer. This provides an ideal opportunity to confer unique metabolic attributes in preparation for disparate TMEs.

Cancer cells can induce and benefit from the formation of metabolic environments that favor tumor growth and impede anti-tumor immunity through nutrient depletion and competition. For example, within the fructose-rich milieu of acute myeloid leukaemia (AML), malignant blasts upregulate GLUT5.^3^ This permits the uptake of fructose, which then enters glycolysis and is subsequently channelled into serine and proline biosynthetic pathways during blast proliferation.^4^ Increased tumour cell metabolism and dysregulated perfusion can create competition for nutrients. In glioblastoma (GBM), tumour cells can extend into regions with limited glucose availability, to which they respond by inducing GLUT5 in an ATF4-dependent manner.^5^ Metabolizing fructose as an alternate fuel source sustains their growth in low-glucose conditions. Although speculative, a shift to fructose may spare glucose for tumour cells undergoing bursts of proliferation or initiating metastasis. A metabolic transition to fructose has also been reported in colorectal cancer and lung adenocarcinoma.^6,7^ Whether other tumours induce GLUT5 to reprogram their metabolism towards fructose is unknown.

GLUTs play an essential role in T cell metabolic reprogramming and anti-tumor function. GLUT1 is the most widely studied glucose transporter in immune cells and has a vital role in T cell effector function.^8,9^ T cells also express GLUT3, which has a higher affinity for glucose than GLUT1. Enforced GLUT3 expression enhanced T cell metabolic fitness and anti-tumour function in a murine model of melanoma.^10^ Mechanistically, GLUT3 promoted glucose uptake and glycogen accumulation in CD8+ T cells. While glycogen provides an important energy reserve, its hydrophilic nature and granular bulk may adversely impact the migratory velocity and hence serial killing activity of CAR-T cells. Glycogen stores are more likely suited to tissue-resident cells such as mucosal-associated invariant T (MAIT) cells.^11^ Several GLUT isoforms require instructive signalling and/or genetic modification to populate the cell membrane in a physiological or experimental context. Which isoform is best suited for adoptive immunotherapy is poorly understood.

We previously showed how energy deficits rather than reductive stress impair T cell function in hostile tumour environments.^12^ In order to optimize fuel selection in CAR-T cells, we considered various fuel types. It is well-established that glucose is required for the energetic and biosynthetic demands of T cell activation.^8,9^ While other fuel sources including the glucose intermediate lactate, or galactose can effectively support mitochondrial energy generation, they are less suited to T cells traversing hostile environments. Lactate exacerbates steep reduced nicotinamide adenine dinucleotide (NADH) redox gradients that occur at hypoxia, and enters glycolysis downstream of phosphoenolpyruvate carboxykinase (PEPCK), an enzyme that produces the glycolytic intermediate phosphoenolpyruvate (PEP). PEP inhibits the sarcoendoplasmic reticulum calcium ATPase (SERCA) to raise cytosolic calcium levels, activate the nuclear factor of activated T-cells (NFAT) family, and support T cell proliferation.^13^ In its most effective form, lactate is transiently elevated to concentrations ranging from 4 mM to 8 mM, and is rapidly oxidized via a cell-cell shuttle.^14^ Conversely, lactate production is sustained in tumours, reaching concentrations up to 40 mM.^15^ Lastly, lactate metabolism is dependent on oxygen, which is severely limited in the core of rapidly growing hypoxic tumours.^16^

Like glucose, galactose is a six-carbon aldose that has potential roles in T cell function.^17^ However, galactose requires ATP “input” to enter glycolysis, which culminates in a lack of net ATP production from the glycolytic phase of its metabolism. Similarly, while fatty acids are abundant in solid tumours and support T cell memory differentiation,^18,19,20,21^ the high energy generating capacity of longer-chain fatty acid subspecies comes with a very high oxygen cost and a delayed rate of ATP synthesis relative to glucose metabolism via glycolysis. Since oxygen can be critically limiting in hypoxic tumours, this would impede fatty acid metabolism.^22^

Given the structural and functional similarities between glucose and fructose, we hypothesize that fructose can be repurposed as a potent fuel source for T cells. The role of fructose in cancer cell metabolism provides a strong premise for our approach: genetically arming CAR-T cells with GLUT5 to facilitate fructose entry, which would allow them to exploit the abundance of fructose as an alternate fuel source in malignancies characterized by a fructose-rich and glucose-poor environment. Importantly, our findings herein on T cell metabolic function, cytotoxicity, and migratory velocity highlight the immediate translational relevance of the fructose/GLUT5 metabolic axis for optimizing fuel selection in T cell-based therapies against cancer, and position GLUT5 as a novel means to selectively fuel CAR-T cells.

## Methods

### Cell Culture

Primary human leukocytes (PBLs) from healthy male and female volunteers were collected at the University of Pennsylvania’s Apheresis Unit. Informed consent was obtained from all participants before collection. All experimental procedures and methods were approved by the University of Pennsylvania Institutional Review Board. T cells were purified at the University’s Human Immunology Core by negative selection using the RosetteSep T cell enrichment cocktail (Stem Cell Technologies). Following isolation, T-cells were cultured in growth medium (GM) comprising RPMI 1640 (Lonza, Basel, Switzerland) supplemented with 10% FBS (HyClone, Logan, UT, USA), 10 mM HEPES, 100 U/ml penicillin G, and 100 μg/ml streptomycin. T cells were activated overnight with 4.5 μm Dynabeads containing immobilized anti-human CD3 and anti-human CD28 (Life Technologies, Carlsbad, CA, USA) at a 3:1 bead to cell ratio. T cells were maintained in culture at a concentration of 0.8–1.0×10^6^ cells/ml by regular counting on a Multisizer III particle counter (Beckman-Coulter). Cells were also counted by flow cytometry using CountBright beads (BD Biosciences, Franklin Lakes, NJ, USA), a viability marker (Viaprobe) and mAbs to either human CD4 or CD8 as described.^23^ Lymphocytes were cultured at 37 °C, 20% O_2_, and 95% humidity with 5% CO_2_ unless otherwise stated. After 10 days, the cells were frozen in aliquots and thawed as needed for experiments.

Human leukaemia cell lines Molm14 and THP-1 were acquired, maintained, and used as previously described.^24,25^

### GLUT 5 Lentiviral Plasmid Construction

pTRPE is a bicistronic lentiviral vector containing a T2A ribosomal skipping sequence that separates two unique coding sequences that are co-translated as separate proteins. pTRPE_eGFP contains the open reading frame for eGFP upstream of T2A permitting an accurate measurement of lentiviral-mediated gene delivery by flow cytometry. The second gene sequence is positioned within AVR11 and Sal1 restriction sites. An expression plasmid for GLUT5 containing built-in AVR11 and Sal1 restriction sites was codon-optimized for mammalian cells. This plasmid was digested with the relevant enzymes, gel purified, and ligated at a 3:1 insert:vector ratio using T4 DNA ligase to create a pTRPE-eGFP_T2A_GLUT5 lentiviral vector encoding eGFP and GLUT5 separated by a T2A self-cleaving peptide and under the transcriptional control of EF-1α. Generation of the pTRPE-eGFP_T2A_anti-CD123CAR; pTRPE-CD20_T2A_anti-CD123CAR; and pTRPE-GLUT5_T2A_anti-CD123CAR lentiviral plasmids followed the same procedure. CAR constructs contained an intracellular 4–1BB signalling domain and an scFv against CD123. as described previously.^24^

### Lentiviral Production

Lentiviral supernatants were generated by transient plasmid transfection of HEK 293T. Producer cells with one of the plasmids whose generation is described above. 293-T cells were initially seeded in T150 flasks and grown to 80% confluence in 25 mL of culture medium (RPMI1640). 90 μl Lipofectamine 2000 DNA transfection reagent was combined with 7 μg pCL-VSVG, 18 μg pRSV-REV, and 18 μg of pGAG-POL (Nature Technology) as well as 15 μg of the chosen plasmid. This mixture was incubated at room temperature for 15 min. DNA-Lipofectamine complexes were then added to the 293-T cells. After 24h, infectious supernatants were sterile filtered through a 0.45-μm syringe tip cellulose acetate filter and collected in a 50 ml conical tube. To pellet the lentivirus, the supernatant was spun in a Thermo Fisher Scientific Centrifuge (LYNX 4000) at 18000 RCF overnight at 4°C. The supernatant was removed, and the lentiviral pellet was resuspended in 1.6 ml of culture medium, aliquoted, and stored at −80°C.

### Lentiviral Infection

Primary human T-cells were activated with Dynabeads as described above. 24h after activation, T cells were seeded at 100,000 cells/well at a concentration of 1×10^6^ cells/ml in a 96-well culture dish. Serial dilutions of lentiviral supernatant over a range of 1:3, 1:9, 1:27, 1:81, 1:243, and 1:729 were performed. Transduced T-cells were grown for 72h to ensure optimal gene expression before comparing transduction efficiencies. The percentage of GFP+/CAR+ cells was determined by flow cytometry, and the corresponding titre was calculated as the number of transforming units (tu)/ml. The titres for all plasmid viral supernatants ranged from 10^6^–10^7^ tu/ml. T cells were infected with lentiviral vectors at multiplicities of infection from 3–5.

### CAR Expression

CAR-T cells were manufactured by activating 10^6^ cells/ml with Dynabeads as described above in RPMI with 10% FBS. Following overnight activation, T cells were lentivirally infected at multiplicities of infection (MOI) ranging from 2–4. Transduced T cells were expanded for 7 days post-activation, debeaded, and stained for CAR expression. 10^6^ cells were washed with PBS, incubated with 10 μl of a pre-titrated biotin-conjugated goat-anti-mouse immunoglobulin G (IgG) F(ab’)2 fragment (Jackson ImmunoResearch) at room temperature for 30 min in fluorescence-activated cell sorting (FACS) buffer consisting of PBS, 1% BSA, and 5 mM EDTA, then washed two times in FACS buffer. Afterwards, the cells were immunostained with streptavidin-PE (BioLegend) for 30 min at room temperature, washed two times in FACS buffer, then fixed in 1% paraformaldehyde in PBS. Non-transduced cells were stained as controls. Positively stained cells were differentiated from background using fluorescence-minus-one controls. Flow cytometry was performed using a LSRII (BD Biosciences, San Jose, CA), and data were analyzed using FlowJo software (Tree Star, Ashland, OR). Cells were evaluated at Day 7 and either frozen, or continued in culture to later resting state.

### Flow Cytometry

At the indicated time following activation, cells were stained with a panel of mAbs (Invitrogen). In all cases, cells were washed with PBS, incubated with antibodies at room temperature for 30 min in fluorescence-activated cell sorting (FACS) buffer consisting of PBS, 1% BSA, and 5 mM EDTA, washed two times in FACS buffer (or stained with secondary if indicated and washed), then fixed in 1% paraformaldehyde. Positively stained cells were differentiated from background using fluorescence-minus-one controls. Flow cytometry was performed using a LSRII (BD Biosciences, San Jose, CA), and data were analyzed using FlowJo software (Tree Star, Ashland, OR).

### Metabolic Function

Mitochondrial function was assessed with XFe96 and XF-Pro instruments (Agilent). T cells were expanded for 7–9 days, then centrifuged at 1,200 rcf for 5 minutes. Cell pellets were resuspended in XF assay medium (non-buffered RPMI 1640) containing 2 mM L-glutamine and either 5 mM glucose or maintained glucose-free. The cells were seeded at 0.2×10^6^ cells per well. During instrument calibration (30 minutes), the cells were switched to a CO_2_-free, 37°C incubator. XF96 assay cartridges were calibrated in accordance with the manufacturer’s instructions. Extracellular acidification rates (ECAR) were measured under basal conditions and following treatment with 10 mM fructose, 1.5 μM oligomycin, 2.5μM BAM15, and 40 nM rotenone with 1 μM antimycin A (XF Cell Mito Stress kit, Seahorse Bioscience).

### Immunocytochemistry

GLUT5 immunostaining was performed using the Alexa Fluor 594 Tyramide SuperBoost kit + streptavidin (ThermoFisher) signal amplification kit. T cells were stimulated with Dynabeads as described above and expanded with regular feeding over several days. Nine days after activation, the cells were seeded in a 96-well poly-D-lysine-coated plate at 5.0×10^4^ cells/well, then centrifuged at 200 rcf for one minute. The cells were fixed in 8% PFA for 10 minutes. Following successive washes in PBS, the cells were treated with blocking buffer containing 5% normal goat serum (ThermoFisher), 0.5% bovine serum albumin (GoldBio), 0.25% Triton X-100 (Fisher BioReagents), and PBS for 1 hour. The cells were incubated overnight at 4°C in blocking buffer with either a mouse anti-GLUT5 antibody (Santa Cruz Biotechnology SC-271055) or corresponding IgG_1_k (R&D Systems IC002R) diluted 1:100 in blocking buffer. The next day, the cells were rinsed with PBS containing 0.2% Tween 20 (PBS-T) and incubated with biotin-conjugated goat anti-mouse IgG (H+L) diluted 1:500 in PBS-T at room temperature for 1 hour. Following successive washes in PBS-T, the cells were incubated overnight in HRP-conjugated streptavidin. The following day, after washes in PBS, the cells were incubated in 1:100 Tyramide solution for 5 min. The reaction was stopped, and the cells were washed in PBS, then PBS-T. Nuclei were counterstained with 1:10000 Hoechst 33342 in PBS-T, after which the cells were washed in PBS-T, and stored in PBS until imaging. Imaging was performed on the BioTek Cytation 5 Cell Imaging Multimode Reader (Agilent).

### Isotope Labelling

For ^13^C-labelled isotope experiments, CAR-T cells were activated with Dynabeads as described above. The cells were expanded with regular counting and feeding on alternate days with standard RPMI culture medium. At Day 8, the CAR-T cells were seeded at a 1:1 effector-to-target ratio with irradiated mRFP+CD123-expressing THP-1 target cells. After clearance of targets was confirmed (using microscopy and flow cytometry), the CAR-T cells were purified using the REAlease CD4/8 (TIL) MicroBead Kit (Miltenyi Biotec). The CAR-T cells were resuspended in RPMI 1640 conditioned with 10% dialyzed FBS (Life Technologies) and supplemented with 10 mM ^13^C_6_ fructose (MilliporeSigma) for 1 hour. Afterwards, the cells were spun down, and both the cell pellets and supernatants were harvested on dry ice for LC/MS analysis.

### Liquid Chromatography-High Resolution Mass Spectrometry

Metabolites and stable isotope incorporation were measured by liquid chromatography-high resolution mass spectrometry adapted from previously published approaches.^26^ Samples were quenched with 1 mL pre-chilled −80°C 80:20 methanol : water (v/v). After vortexing for 1 min samples were retuned to −80°C 30 min, centrifuged 18000 × g 10 min at 4°C, and supernatant was transferred to a 96-well plate and evaporated to dryness under nitrogen gas. Samples were reconstituted in 100 μL then 2 μL of the sample kept at 4 °C was injected from an autosampler onto a 25 °C ZIC-pHILIC 150 × 2.1 mm 5 μm particle size column (EMD Millipore) with a ZIC-pHILIC 20 × 2.1 guard column in a Vanquish Duo UHPLC System (Thermo Fisher Scientific). Chromatography conditions were as follows: buffer A was acetonitrile; buffer B was 20 mM ammonium carbonate, 0.1% (v/v) ammonium hydroxide in water without pH adjustment, with a gradient of 0.5 min at 20% A then a linear gradient from 20% to 80% B; 20–20.5 min: from 80% to 20% B; 20.5–28 min: hold at 20% B at a 0.150 mL/min flow rate. The column elute was introduced to a Q Exactive Plus with a HESI II probe operating in polarity switching mode with full scans from 70–1000 m/z with an insource fragmentation energy of 1. Instruments were controlled via XCalibur 4.1 and data was analyzed on Tracefinder 5.1 using a 5ppm window from the predominant M-H negative ion. For isotope tracing, isotopologue enrichment was calculated using FluxFix.^27^

### Anti-CD123 CAR-T Cell Cytotoxicity Assay

Tumour target cells (THP-1) were plated on a 96-well plate (Corning) at 3×10^4^ cells/well in 100 μl of RPMI-based GM described above supplemented with 10% dialyzed FBS and either 10 mM glucose, or 10 mM fructose, or maintained glucose-free. Cells were incubated at 37°C overnight. Pre-treatment cell viability of the target cells was monitored by xCELLigence RTCA eSight machine (Agilent) for 18 to 20 h. Day 9 CAR-T cells were serum starved for 30 minutes in serum-free GM, then resuspended in the corresponding GM and added to the target cells at an effector-to-target ratio of 0.25 in a total volume of 100 μl. The cell viability was monitored with the xCELLigence RTCA eSight for 90–120 h. The cell viability of each assay well was normalized to the last target total integrated fluorescence intensity of pre-treatment incubation. Percent cytolysis was plotted as the percent difference of total integrated fluorescence intensity between the normalized baseline at the start of the assay and the last time point. For fluorescent imaging, both target and effector cells expressed fluorescent proteins: mRFP for targets, and eGFP for effectors. Bright field images and fluorescent (green, red) images were taken with xCELLigence RTCA eSight. T cell-mediated cytotoxicity assays were performed with three technical replicates for *in vitro* samples. Four independent experiments were performed with separate donors to confirm CAR-GLUT5-mediated cytotoxic activity.

### T Cell Velocity Measurements

Tumour target cells (EM-meso cells engineered to express a CD123-GFP fusion protein) were seeded at 1.2×10^4^ cells/well. Pre-treatment cell viability of the target cells was monitored by Nanolive 3D Cell Explorer 96focus machine (Nanolive SA) for a few measurement cycles (around 8 minutes each). T cells were transduced with CD20-anti-CD123 CAR versus GLUT5-anti-CD123CAR and expanded for 7 days until restdown. These cells were thawed and resuspended in glucose-free RPMI supplemented with 2% Physiologix (Nucleus Biologics) and conditioned with/without 10mM fructose. After normalizing for CAR expression levels, they were added to EM-meso target cells previously engineered to express CD123 at an effector-to-target ratio of 1.25:1. The cell viability was monitored with the 3D Cell Explorer 96focus for 41 measurement cycles (around 5 h). The Nanolive label-free system was used observe the motility of the CAR-T cells in real-time. Using the proprietary AI, the generated images and videos were analyzed to highlight the effector cells and measure their velocity (in μm/s), which was computed as the mean velocity for all effectors. The mean velocity was normalized to the velocity recorded at the first measurement cycle, and was plotted over time. Bright field images and videos were taken with 3D Cell Explorer 96focus.

### *In Vivo* Xenograft Model

A xenograft model of AML was used in this study as previously described.^24^ Briefly, 6–10-week-old male NOD-SCID γc−/− (NSG) mice were obtained from Jackson Laboratories (Bar Harbor, ME, USA) or bred in-house under a protocol approved by the Institutional Animal Care and Use Committees (IACUC) of the University of Pennsylvania. The mice were injected via tail vein with 1.0×10^6^ Molm14 cells expressing click beetle green (CBG) luciferase in 0.1 ml sterile PBS. On Day 6, anesthetized mice were imaged using a Xenogen IVIS Spectrum system (Caliper Life Science) with an intraperitoneal injection of D-luciferin (150 mg/kg; Caliper Life Sciences). BLI was quantified using Living Image 4.4 (PerkinElmer) and animals normalized into groups. On Day 7, T cells (anti-CD123 CAR-Ts co-expressing either CD20 or GLUT5) were injected IV at the indicated dose of 0.8×10^6^. Non-transduced T cells (NTD) served as the control. Tumour growth was monitored by bioluminescent imaging (Xenogen Spectrum system; Living Image v4.7.4 software; Advanced Molecular Vision, Grantham, UK). Peripheral blood was obtained by retro-orbital bleeding in an EDTA-coated tube, and was examined fresh for levels of T cell engraftment and phenotypes by flow cytometry using BD Trucount (BD Biosciences). Animals were euthanized at the end of the experiment before showing signs of toxicity and before reaching signals higher 1×10^10^ p/s total flux per mouse (in accordance with our IACUC protocols).

### Statistical Analyses

To determine statistical significance, we analyzed the mean data obtained from two groups using paired Student’s t tests. When comparing differences across multiple groups with a single independent variable, a one-way ANOVA with Newman-Keuls post-test comparisons were performed. Data from multiple groups with two independent variables were analyzed by two-way ANOVA using the Newman-Keuls post-test comparisons method. CAR-T cell functional analyses were performed using GraphPad Prism version 8.1.6e (GraphPad Software). For all tests, the significance of the difference between means was accepted as <0.05 level.

## Results

### GLUT5 Induction is an Important Feature of Cancer Cell Metabolism

**Figure 1.**
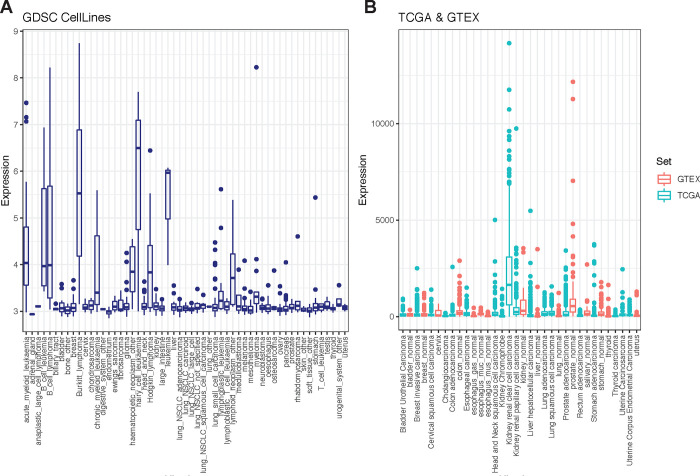
GLUT5 induction is a metabolic adaptation observed in several tumour cells A) Gene expression of GLUT5 across 1000 human cancer cell lines found in the Genomics of Drug Sensitivity in Cancer (GDSC) database. B) GLUT5 gene expression in various cancer samples (TCGA) and normal human tissue (GTEX) from cBioportal. Tissue is ordered by highest mean expression.

The role of glucose in T cell activation, proliferation, and differentiation has been well explored.^8,9^ Fructose is a monosaccharide like glucose which led us to consider can it be leveraged as an alternate fuel source? One key distinguishing feature between these functional isomers lies in their cyclic form: fructose is a ketose that forms a five member ring, while glucose is an aldose that forms a six member ring. This structural diversity explains the ability of some GLUT isoforms (for example GLUT1, 3, and 4) to preferentially transport glucose over fructose, and underscores the need for a specialized transporter (GLUT5) to shunt fructose across the cell membrane. Once “captured” inside the cell, we hypothesized that fructose can be an alternate fuel source for T cells, replacing their need for glucose. GLUT5 is endogenously expressed in enterocytes, hepatocytes, kidney epithelial cells, adipocytes, and skeletal myoblasts.^28^ Increasing evidence suggests that some tumor cells - such as those of AML and GBM - induce GLUT5 to exploit fructose metabolism and spare glucose. To determine how broadly this adaptation extends, we screened the GLUT5 protein coding gene, SLC2A5, in datasets registered in the Genomics of Drug Sensitivity in Cancer (GDSC), Cancer Genome Atlas (TCGA) and the Genotype-Tissue Expression (GTEx) Portal. We show that GLUT5 is a metabolic adaptation that extends to several other tumours (Fig. 1A&B). Tumour cells and activated T cells share several metabolic features. This provides strong support for our premise that the fructose/GLUT5 metabolic axis can be leveraged to optimize CAR-T cell metabolic function when fructose is enriched, or when glucose is scarce.

### Fructose Metabolism Sustains Maximal Glycolytic Capacity and ATP Replenishment Rates for T Cells in Glucose-free Conditions

**Figure 2.**
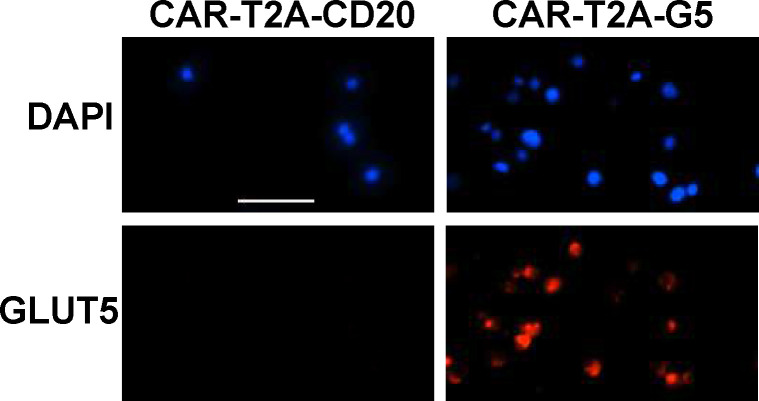
Lentiviral-mediated expression of GLUT5 in T cells GLUT5 expression following lentiviral transduction was determined by immunostaining with an antibody against GLUT5. Nuclei were counterstained with the fluorescent dye Hoechst. No immunostaining was observed with an IgG control (data not shown). Representative data from multiple experiments are shown.

**Figure 3.**
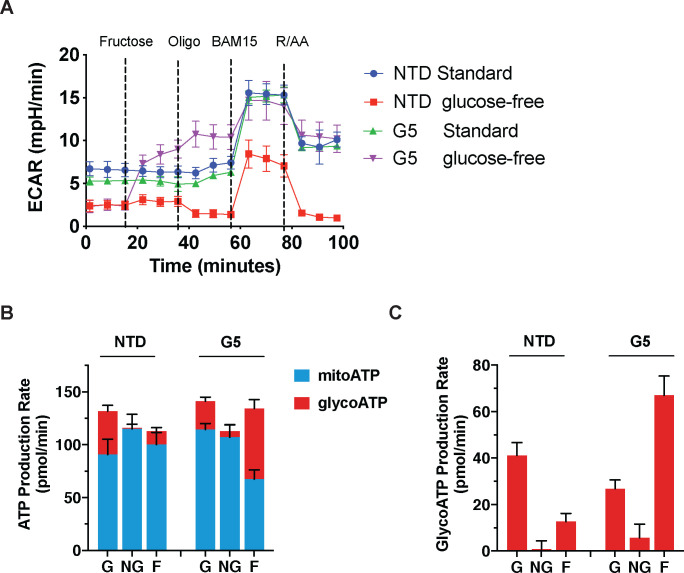
GLUT5 confers metabolic flexibility in low glucose conditions A) Following overnight stimulation with anti-CD3/CD28 Dynabeads, primary human T cells were infected with GLUT5 lentiviral supernatants and expanded for 9 days (NTD: non-transduced controls). These cells were switched to standard (Std) or glucose-free Seahorse assay medium. Metabolic responses to 10 mM fructose, oligomycin, BAM15, as well as rotenone and antimycin A were measured by Seahorse Assay. Representative data (mean +/− SEM) from 3 independent experiments with separate donors are shown. B) Estimated ATP production rates in glucose (G), no-glucose (NG), and fructose (F) conditions. Mitochondrial ATP production (mitoATP) is represented by the blue bar fraction for each group, while glycolytic ATP production (glycoATP) is represented by the red bar fraction for each group. Black error bars indicate SEM. C) Bar graph of glycoATP alone, stylized in the same manner as Panel c. Representative data (mean ± SEM) from several independent experiments with separate donors are shown.

To study the impact of GLUT5 in T cells, we transduced primary human lymphocytes with wild-type GLUT5 lentiviral supernatants. We confirmed expression by immunocytochemistry (Fig. 2). As metabolic reprogramming is an important determinant of T cell function, we examined the metabolic properties of GLUT5-expressing T cells by extracellular flux analysis. Non-transduced (NTD) control and GLUT5-expressing cells were cultured in standard medium for 9 days, before switching to XF RPMI assay medium with/without glucose for the assay. After establishing the steady state baseline metabolic properties, we injected fructose (to a final concentration of 10 mM), followed by the three standardized injections (oligomycin, BAM15, and rotenone/antimycin A). GLUT5-expressing cells respond to fructose with a robust increase in glycolytic activity; a feature accentuated by oligomycin treatment (Fig. 3A). Importantly, glycolytic response rates achieved with fructose/GLUT5 matched those of T cells exposed to XF assay medium containing glucose. We also provide novel evidence that fructose expedites ATP replenishment (Fig. 3B), primarily by glycolysis (Fig. 3C). These findings show that GLUT5-expressing T cells can bypass their dependence on glucose and instead switch to fructose as an alternative carbon source to achieve optimal energy-generating capacity.

### CAR-T Cells Metabolize Fructose through Glycolysis and the TCA Cycle

**Figure 4.**
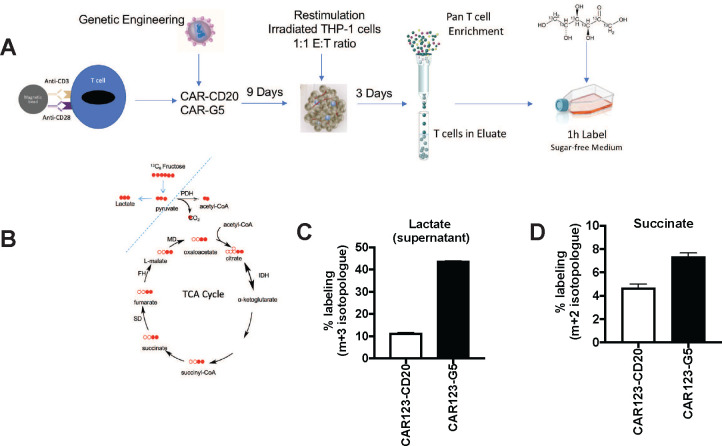
The fructose/GLUT5 metabolic axis supports glycolysis and TCA cycle anaplerosis A) Scheme of experimental design. Activated T cells were transduced with CAR123 expressing either CD20 (control gene) or GLUT5. These cells were expanded for 9 days. At restdown, they were co-cultured with irradiated target cells (mRFP+ THP-1) at a 1:1 E:T ratio. After 3 days, T cells were column purified and transferred into medium containing 10 mM ^13^C-labelled fructose. After one hour, cells and supernatants were harvested for LC-MS. B) Filled red circles indicate ^13^C atoms, unfilled red circles ^12^C atoms into descendent metabolites. C) Funnelling of fructose was quantified as % labelling (y-axis; m+3 isotopologue for lactate, m+2 for succinate). Representative data (mean ± SEM) from two independent experiments with separate donors are shown.

To understand how fructose supports metabolism in GLUT5-expressing T cells, we traced the fate of ^13^C_6_ fructose in central metabolic pathways supporting energy generation, nucleotide production, and lipid biosynthesis. We were particularly interested to see how fructose metabolism in T cells compares to leukemic cell lines, which effectively funnelled fructose into serine and glycine biosynthesis through the serine synthesis pathway.^4^ As seen in the schematic presented in Fig. 4A, anti-CD123 CAR-T cells, co-expressing either CD20 or GLUT5, were cultured for 10 days, then restimulated though their CAR with irradiated mRFP+ THP-1 cells. Following target clearance (as confirmed by microscopy and flow cytometry), T cells were column purified and transferred to media spiked with either unlabelled or isotopically-labelled ^13^C_6_ fructose. After 1 hour, the cells and the supernatants were harvested for LC/MS analysis. Cytoplasmic and mitochondrial routes of fructose metabolism are depicted in Fig. 4B. We found that 44% of lactate accumulation was derived from fructose, which provides conclusive evidence that T cells can effectively metabolize fructose as a fuel source (Fig. 4C). This is noteworthy as other cell types including hepatocytes require fructokinase (lacking in T cells) to initiate fructose metabolism.^29^ Our findings reveal that fructose supports TCA anaplerosis, based on the 1.6 fold increase in intracellular succinate labelling (Fig. 4D). This contrasts the fate of fructose in AML cells, where fructose metabolism is mainly restricted to the cytoplasm.^4^ Our findings highlight fructose as a very robust fuel source that readily undergoes glycolytic metabolism and mitochondrial oxidation in GLUT5-expressing CAR-T cells.

### The GLUT5/Fructose Axis Rescues the Cytolytic Defect of Standard CAR-T Cells in the Absence of Glucose

**Figure 5.**
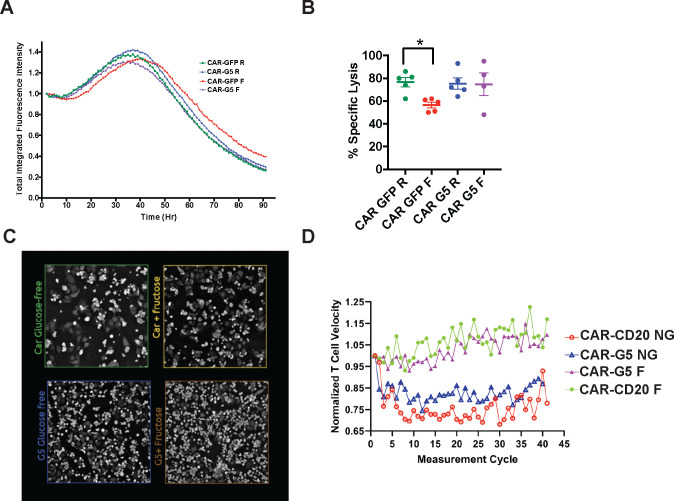
GLUT5 rescues CAR T cell cytotoxicity in low-glucose conditions Activated T cells were transduced with CAR123 co-transduced with GFP or GLUT5. These cells were expanded in standard medium for 8 days. At restdown, they were co-cultured with target cells (mRFP+ THP-1) at an effector:target ratio of 1:1. Cytolytic activity was measured in culture medium with/without glucose, ± fructose using eSight RTCA impedance. A) Normalized total integrated fluorescent intensity (y-axis) was measured over time in hours (Hr, x-axis). Representative images from 4 independent experiments with separate donors are shown. B) Without glucose, CAR GFP has significantly diminished cytolytic activity (* p<0.05 by t-test analysis). GLUT5 restores CAR T cell cytolytic activity when fructose replaces glucose in culture medium. Mean ± SEM values from 4–5 independent experiments with separate donors are shown. R: RPMI standard medium; F: glucose-free RPMI medium supplemented with 10mM fructose.

Competition for nutrients can induce cellular energy deficits that ultimately impede CAR-T cell cytolytic function.^12,22^ We note however, that fructose is abundant in the bone marrow and periphery in AML.^3^ To determine the functional significance of our findings, we investigated if fructose supports CAR123-GLUT5 cytotoxicity using xCELLigence RTCA eSight technology. CAR123-CAR-T cells co-expressing either eGFP or GLUT5 co-expressed, were co-cultured with mRFP+ THP-1 target cells, in medium with glucose or with fructose. Specific lysis was measured by the loss of total integrated red fluorescence over the course of the assay (Fig. 5A). Under nutrient-restricted conditions (absence of glucose), CAR123-eGFP cytolytic activity was impaired by approximately 20% (Fig. 5B). GLUT5 completely rescued this cytolytic defect, thereby providing compelling evidence that the inherent fuel flexibility of GLUT5-expressing CAR-T cells renders them less sensitive to glucose depletion in tumours. This proof-of-concept model shows that GLUT5 confers superior resilience for CAR-Ts targeted against AML.

### GLUT5-CAR-T Cells Demonstrate Enhanced Motility while Consuming Fructose

T cell migration is an essential pre-requisite for immunologic synapse formation and cytolysis. Following cytolysis, CAR-Ts rapidly advance to their next target and re-initiate synapse formation. Considering the “hit-and-run” nature of serial killing, we investigated how the fructose/GLUT5 axis impacts T cell motility. Using the Nanolive 3D Cell Explorer, we measured CAR-T cell velocity in response to the EM-meso an adherent cell line that was engineered to express CD123. This cell line originates from a mesothelioma pleural effusion, and has been extensively used to assess CAR-T cell function.^12,30,31^ CAR-T cell velocity was tracked in real time (μm/s), then quantified by Nanolive AI analysis (Fig. 5C). We provide the first quantitative evidence that CAR-T cell velocity is impaired when glucose is limited (Fig. 5D). Access to fructose rescued deficits in cell velocity regardless of GLUT5 expression. This likely reflects the non-physiologic conditions (fructose concentration of 10 mM) established in this assay. At this concentration, we observed ^13^C_6_ fructose labels of lactate and succinate, albeit at lower levels than those measured in GLUT5-expressing T cells (Fig. 4C&D). Interestingly, T cells express low levels of GLUT8, which may contribute to these responses.^8^ Taken together, our findings reinforce how fructose and GLUT5 improve CAR-T cell functional competence: by potentiating both the cytolytic and the migratory activities of CAR-Ts against cancer.

### GLUT5 Enhances CAR-T Cell Anti-Tumour Function *In Vivo*

**Figure 6.**
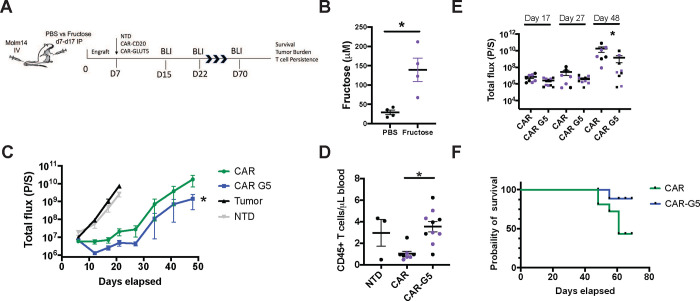
The fructose/GLUT5 metabolic axis supports CAR T cell migratory velocity during serial killing Activated T cells were transduced with CAR123 co-expressing CD20 or GLUT5. These cells were expanded in standard medium for 7 days. After thaw, these cells were co-cultured with CD123+ target cells at a 1.25:1 CAR+ E:T ratio in glucose-free RPMI supplemented with 2% Physiologix, 2mM glutamine, with/without 10mM fructose. Data are Mean ± SEM values. Automated live cell imaging as well as analysis was performed by Nanolive technology.

To determine if GLUT5 conferred a metabolic benefit to CAR-T cells *in vivo*, we evaluated the anti-tumour function of GLUT5-expressing CAR-T cells in our well-established preclinical model of AML (Fig. 6A).^24^ Immunodeficient mice were transplanted with CBG-expressing Molm14 cells. Tumour cell engraftment was confirmed 6 days later by bioluminescence. Initial tumour burden was similar across all groups. We compared the potency of CAR-T cells (anti-CD123-CD20 vs. anti-CD123-GLUT5) by infusing a low dose of 0.5×10^6^ CAR+ T cells intravenously (IV) and measuring tumour burden by bioluminescence at regular intervals. A “stress test” model integrating a low dose of CAR-Ts is increasingly recognized as an important experimental parameter to provide insights into potency.^32,33^ In this model, CAR-T cells enter a hypofunctional state and lose durability for poorly understood reasons. This occurs independent of antigen escape/loss. The unique metabolic nature of AML suggests metabolic dysfunction and energy deficits may contribute to CAR-T cell dysfunction. Thus, our model recapitulates the fate of anti-CD123-CAR-T cells in clinical settings, and will provide insight into the challenges impeding their efficacy. As expected, untreated Molm14 cells grew exponentially over time and control T cells (NTD) had no impact on tumour cell growth (Fig. 6C). We provide evidence that GLUT5-expressing CAR-Ts significantly outperformed standard CAR-T cells throughout this model (Fig. 6D).

From Day 7 to Day 17, a subset of mice were intraperitoneally (IP) injected either with sterile PBS or 10% w/v fructose in PBS. This intervention increased serum fructose concentrations by two-fold (reaching approximately 150 μM; Fig. 6B). At this level, there was no intended benefit to CAR-T cells. Yet, we found that GLUT5 was sufficient to enhance CAR-T cell potency without any fructose intervention (Fig. 6C&E). Thus, therapeutic outcome is dependent on the metabolic conditions encountered. Specifically, the relative abundance of glucose vs fructose will determine GLUT5 transporter activity and, ultimately, metabolic fate. Unless fructose levels are dramatically increased, our findings imply that GLUT5 enhances the cells’ avidity for glucose by serving as an effective glucose transporter. During the experiment, we collected peripheral blood samples from mice treated with CAR-Ts. We measured 3x more circulating human T cells in the CAR123-GLUT5 group compared to the experimental control CAR123-CD20 (Fig. 6D). The increased persistence of GLUT5-expressing CAR-Ts contributed to a 40% higher survival rate than those receiving standard CAR-Ts (Fig. 6F).

### Potential Sources of Fructose in the AML Bone Marrow

**Figure 7.**
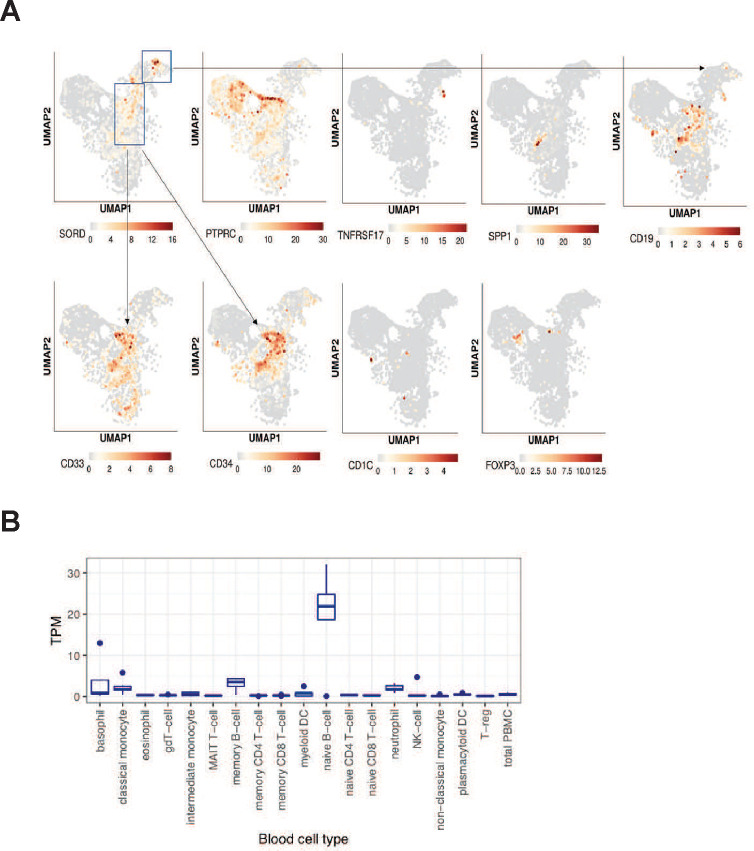
GLUT5 potentiates CD123-CAR T cells anti-tumour function in vivo A) Adult NSG mice were infused by tail vein injection with 1 × 10^6^ luciferase-expressing MOLM14 tumour cells (day 0). On day 7, the mice received 0.5 × 10^6^ CAR+ T cells in PBS via tail vein. T cells expressed tumour targeting CD123 CAR constructs (CAR) co-expressing CD20 or GLUT5. Non-transduced (NTD) T cells were used as a control. The mice were intraperitoneally injected with either sterile PBS (black dots) or with 10% w/v fructose in PBS (purple dots). Treatments are stylized the same in all panels. B) Absolute fructose levels 30 min following IP injection are shown (n=4 mice per condition; p<0.05 by Student t-test analysis). C) Serial quantification of tumour burden in Molm14 tumour-bearing mice treated with CAR T cells. Data are means ± SEM from starting cohorts (n=12 for tumour alone; n= 5 for NTD; n=8 mice for CAR-CD20; and n=10 for CAR-GLUT5). Tumor volume was compared across CAR groups by two-way ANOVA with Holm-Sidak post hoc analysis. * indicates tumor volume is statistically lower in CAR G5 at day 48 post T cell injection (p<0.05). D) Blood harvested on day 13 by retro-orbital puncture was stained with an antibody against human CD45 in TrueCount tubes to detect adoptively transferred T cells. Data are CD45+T cells per μL blood. Individual mice plus cohort means ± SEM are shown. Circulating T cells are significantly enhanced in CAR GLUT5 relative to CAR GFP (p<0.05 by one way ANOVA with Newman-Keuls multiple comparisons test). E) Tumor volume was compared across groups by one way ANOVA with Newman-Keuls multiple comparisons test. * indicates tumour volume is statistically less in CAR− GLUT5 from CAR-CD20 GLUT5 (p<0.05). Individual mice plus cohort means ± SEM are shown for days 17, 27, and 48. F) Survival proportions of Molm14 tumour-bearing mice in each treatment cohort. Mice with BLI measurements over 1×10^10^ photon flux (p/s) were sacrificed. Analysis with log rank (Mantel-Cox) testing demonstrated that GLUT5 is significantly different than the standard CAR (p<0.05). Two independent experiments with GLUT5-expressing, CD123-specific CAR T cells were performed. B cells are a potential source of fructose in AML tumor environment A) UMAP projection shows lineage-specific SORD expression in the bone marrow of AML patients. Representative data from 5 AML patients undergoing CD123 CAR therapy at Penn are shown. Cells are colour-coded to distinguish SORD expression across lineages. Phenotypic markers distinguish hematopoietic (PTPRC=CD45), plasma cells (TNFRSF17=BCMA), and B cells (CD19). SORD is most abundant in CD19+ B cells. B) GLUT5 gene expression in blood cells at various stages of their development. Data was generated from the cBioportal resource.

Physiologic serum fructose levels range from 20–150 μM.^34^ In the context of AML, fructose accumulates in the bone marrow, reaching concentrations of 2 mM, 5 mM, and in some cases up to 8 mM. Systemic levels also increase, reaching up to 1 mM in the periphery.^3^ Implicit in this observation is that fructose is produced by cells present in the bone marrow, and diffuses into the larger blood volume of the periphery. Importantly, the source of fructose production has not been described. As sorbitol dehydrogenase (encoded by SORD) synthesizes fructose at the end of the polyol pathway, we profiled SORD abundance in human bone marrow using single cell transcriptomic data generated from the anti-CD123-CAR-T cell clinical trial (NCT04106076) performed at the University of Pennsylvania.^35^ Uniform manifold approximation and projection (UMAP) visualization revealed SORD expression in AML blasts (CD33+ and CD34+; Fig. 7A). This was expected as they account for at least 20% of all the cells in the leukemic bone marrow.^36^ Interestingly, the highest SORD transcript levels were mapped to a non-AML cell population. These cells are haematopoietic in origin (CD45/PTPRC+ cells). Our findings exclude plasma cells as the SORD+ cells lack BCMA (TNFRSF17). Expression of CD19 without CD20 or CD22 positions it as a B cell population at the more primitive stage of its differentiation program. Data from the human protein atlas reveals that naïve B cells express high levels of GLUT5 which could facilitative fructose diffusion across the cell membrane. (Fig. 7B).

## Discussion

We engineered CAR-T cells to express GLUT5 to intercept metabolic adaptations unique to cancer cells encountering glucose restriction. Given the selectively of GLUT5 for fructose, our goal was to potentiate CAR-T function in the fructose-rich environment of AML.^3,4^ We established that GLUT5 supports maximal glycolytic capacity and expedites ATP replenishment using fructose as the sole fuel source (Fig. 3). In AML, malignant blasts primarily channel fructose to the serine synthesis pathway (SSP) for biomass accrual. Fructose can also be converted to lactate in cells undergoing aerobic glycolysis (Fig. 4C). Here, we identify a unique feature of fructose metabolism in GLUT5-engineered T cells. Using ^13^C tracer technology, we show that ^13^C_6_ fructose enters the mitochondria, where it supports TCA cycle anaplerosis (Fig. 4D). Our findings position the GLUT5/fructose metabolic axis as a novel strategy to overcome competition for glucose in appropriate tumour environments, and support optimal mitochondrial function for long-lasting immunosurveillance.

In GBM, nutrient stress from low glucose conditions induces an ATF-4-dependent fructolytic response (expression of GLUT5 and aldolase B). We provide evidence that several other tumours express GLUT5 (Fig. 1A&B). Implicit in this observation is that GLUT5 induction is an understudied adaptation of tumour cell metabolism. This can be exploited to potentiate CAR T cell metabolic fitness and anti-tumour function. In hepatocytes, ketohexokinases (KHK-A and C) initiate fructose metabolism by phosphorylating fructose to fructose-1-phosphate. Despite lacking endogenous expression of KHK, T cells underwent complete metabolism of ^13^C_6_ fructose to lactate. These findings show that T cells are primed to use fructose as an important fuel source once there is no limitation to its uptake. Given our findings, we hypothesize that HK2 has sufficient flexibility to metabolize either glucose or fructose. Co-expressing KHK had no observable benefit on fructose metabolism in GLUT5-exressing T cells (data not shown).

Redirecting T cells towards alternative fuel sources is an important strategy to counter energy deficits experienced in the TME. We hypothesized that GLUT5-expressing CAR-T cells would benefit from enhanced fructose availability in our xenograft model of AML. We choose daily intraperitoneal injections to raise systemic fructose levels during the initial phase of the xenograft model for several reasons. Fructose infusions have been safely applied to directly support metabolic activity in skeletal muscle cells (which endogenously express GLUT5) during endurance events that deplete plasma glucose.^37^ Parenteral routes bypass the post-prandial conversion to lactate that occurs in splanchnic organs.^38,39,40^ Clinically, we note that KHK inhibitors could be repurposed to raise plasma fructose in a combinatorial manner with GLUT5-expressing CAR-Ts against cancer. Remarkably, we found that GLUT5-expressing CAR-Ts demonstrated superior anti-tumour function independent of fructose (Fig. 6C–F). This unexpected outcome can be explained by an enhanced ability of CAR-T cells to metabolize glucose via GLUT5. This culminated from the relative abundances achieved in our model (fructose: 150 μM; glucose: 5 mM). Notwithstanding its source of fuel, arming CAR-T cells with GLUT5 translates to significantly enhanced anti-tumour function and survival *in vivo*. The enhanced durable efficacy of GLUT5-expressing CAR-Ts highlights the vital role that glucose plays in CAR-T cell differentiation. We propose that GLUT5 confers a hybrid-like CAR-T design with engineered fuel flexibility: enhanced affinity for glucose when glucose levels are in the mM range; and the ability to switch to fructose when the inverse scenario occurs (higher fructose relative to glucose such as what occurs in AML). This dual benefit supports CAR-T cell efficacy *in vivo*.

Cytolysis involves a multi-step process of migration, adhesion, synapse formation, followed by the release of cytolytic effector molecules and pro-inflammatory cytokines. What fuels individual aspects of cytolysis - especially the migratory component of serial killing is unknown. We show that the fructose/GLUT5 metabolic axis restores maximal migratory velocity in tumour cell : CAR-T cell co-cultures (Fig. 5D). We propose that GLUT5 coordinates fructose uptake when glucose is scarce to support the energy demands of migration. Future efforts are underway to uncouple migration from cytolysis, in order to determine their relative energy cost during serial killing. Our findings suggest that reprogramming metabolism via GLUT5 provides an actionable strategy that may benefit T cell-based therapies against solid tumours where the metabolic nature of the TME impedes CAR-T cell killing.

We recognize that AML blasts and CD123-CAR-T cells engineered to express GLUT5 may compete for fructose in the bone marrow. As GLUT8 also displays high affinity for fructose, it emerges as an important candidate to inspire similar approaches. Intuitively, select inhibitors of GLUT5 such as MSNBA (N-[4-(methylsulfonyl)-2-nitrophenyl]-1,3-benzodioxol-5-amine; K_i_ of 3.2 ± 0.4 μM) can be combined with GLUT8-expressing CAR-T cells to bypass competition for fructose in AML.^41^

Extracellular fructose levels are enhanced within the bone marrow in AML. This implies that fructose is produced locally within the bone marrow niche. AML blasts account for 20% of the cells found in the bone marrow, which positions them as the most obvious source of fructose production. However, our findings suggest that AML cells may also induce their surrounding cells to produce fructose and release it into the local environment. scRNA seq data from the CAR-123 trial for AML revealed high SORD expression in AML blasts as well as (non-malignant) immature B lymphocytes (Fig. 7A). This restricted expression implicates B cells as a potential source of fructose in AML. Together, these unexpected findings suggest that AML blasts may co-opt fructose produced locally by B cells. Within the Human Protein Atlas, naïve B cells express high levels of GLUT5 (Fig. 7B), which raises questions surrounding the impact GLUT transporters have on fate/commitment in the B cell lineage. Our novel findings will inspire future studies to precisely determine the role of the fructose/GLUT5 metabolic axis in leukemic cell neoplastic transformation and stem cell biology.

In summary, we show that T cell dependency on glucose can be mitigated by facilitating the metabolism of fructose, a closely related functional isomer. Our findings provide an important advance in the clinical applications of CAR-T therapy against AML, and potentially other tumours where fructose is abundant. Consistent with its intended purpose, GLUT5 supports T cell proliferation in fructose-containing culture medium that lacks glucose. GLUT5 rescues metabolic fitness by conferring a unique flexibility for fructose. Expressing glucose transporters to optimize fuel selection, expedite ATP replenishment, and bolster anti-tumour function has been fraught with challenges. Here, we show that GLUT5 is an ideal candidate with immediate translational relevance, including CAR-Ts against AML. Future directions will likely see an expansion of this platform to CAR-Ts targeting solid tumours.
